# Diseases of the Lungs

**Published:** 1895-07-06

**Authors:** 


### Progress in Medicine.

### DISEASES OF THE LUNGS.

**Pneumonia.**—Washbourn,[1] at the Pathological Society, confirmed the common view that Fränkel's pneumococcus is really the active agent in lobar pneumonia, as well as in many cases of pleurisy, meningitis, otitis, and lobular pneumonia. The difficulty of cultivating it is great, from its delicacy and variable virulence. It succeeds best on a thin layer of blood over agar jelly. Immunity for a short time can be produced by injection of the serum of immunised animals and of toxic products. The *Lancet* of January 26th summarised the results of curative treatment of late by similar methods. Thus the brothers Klemperer had good results in more than twenty cases, while Foa, Jansen, Scolia, and others reported like successes. On the whole, the disease was shortened and convalescence was rapid.

A. E. Wright argued that pneumonia was primarily a local inflammatory process, and thus differed first from septicæmia and from the "intoxication process" of diphtheria, where the bacilli lie on the outer or inner surface of the body, and the toxins alone enter the circulation.[2] The cocci invade the lung in pneumonia, and the immigration of the white corpuscles into it is comparable to what occurs in an abscess. The crisis marks the end of another stage of pneumonia—that of true septicæmia. The white corpuscles have multiplied and cleared the blood of the invading cocci, and then disappear into the spleen. A similar process is seen in relapsing fever, but it is not proved that the cocci are ever numerous in the blood during pneumonia before the crisis. The process of resolution in the caseous lung is remarkable for the removal of the great mass of nucleo-albumen produced from the disintegrated white corpuscles without the occurrence of intra-vascular clotting. It appears to be broken up into uric acid and other substances. Indeed, uric acid is usually, as Horbaczewski showed, a product of broken-down white corpuscles, and not of nitrogenous metabolism. The leucocytosis so often noticed has been examined by Billings.[3] It is usually present in cases taking a favourable course, and the excess of white corpuscles disappears shortly after the crisis has occurred. In certain fatal cases and in very mild ones it may be entirely absent. Talamon[4] thought that whatever is the pathology, the contagiousness of the disease cannot be doubted, and that herpes is of little significance, but on the whole of favourable prognosis. Bozzolo has discussed[5] the cases of paralysis without discoverable lesions which are occasionally met with, and which may be due to grave disturbances of the cerebral circulation. We may compare certain early paralyses in syphilis with these cases in pneumonia and other febrile disorders according to J. Hutchinson, since both are probably produced by some toxin like that of diphtheria. Three cases of pneumonia arising after a blow have been related by Mongour,[6] while Meunier discussed[7] the ætiology of those which follow injury, inflammation, or compression of the vagus. Such lesions, he thought, act by lessening the resistance of the lung to microbial invasion. A similar effect is produced by the presence of the microbe of furunculosis, staphylococcus aureus, in the body, which greatly increases the virulence of the pneumococcus.[8] F. W. Garber has argued that it seems reasonable to believe that tissue soil is as much influenced by atmospheric conditions for the growth of these organisms as the earth is for the growth of higher forms of vegetation,[9] and he has also given instances showing the infectiousness at times of pneumonia. Similar cases have been collected by L. Gillespie, but we notice that influenza was prevalent at the time, and may possibly account for the attacks.[10] Surgeon-Major Duncan, too, has recently discussed an outbreak in the 23rd Pioneers, which practically ceased after disinfection of their camp.[11] As a complication of pneumonia arthritis is occasionally met with, and in a case of Meunier's both the pneumo and streptococci were found in the affected knee.[12] E. Fränkel has examined a large number of cases of pneumonia where albuminuria was present.[13] The typical pneumonia kidney was remarkable by its absence. There was rarely any interstitial change, but generally acute toxic ones in the parenchyma. Parotiditis does not appear to be always a complication due to the specific microbe, but may occur, according to J. Hobbs, during the course of the disease from external causes.[14] Though pneumonia during pregnancy is very fatal, a case affecting both lungs, together with heart disease and empyæma, was reported as recovering by F. Hawkins.[15] As to treatment, Leech reviewed various methods which have been tried at different times. Neither active nor expectant treatment has given reliable results. The application of cold, the use of veratrum, or of digitalis, have each of them value in certain cases, but no single method is of general applicability.[16] The use of oxygen inhalation has been much discussed, and J. W. Russell has stated[17] that though much over-rated, it might be of real value when very little air enters the lung and long-continued coma is met with. The treatment by very large doses of digitalis was upheld by Petresco, who claimed that it produced the lowest mortality, and that one to two drachms of the leaves might be given with advantage daily in infusion. He treated[18] 155 cases, with a mortality of only 1·2 per cent. Fickl, Hoepfel, and Strizover reported 132 more cases with good results, while Lowenthal and Renier found the danger of poisoning considerable and the effect on the disease little, though Lowenthal admitted that a single dose of a drachm produces at times extraordinarily good results. In criticising these reports Hare believed that Petresco's success was due to the sedative action of the doses used. After the crisis he would rely instead on strychnia and belladonna.[19] Ivanoff praised injections of camphor in oil in severe cases, and camphor with antipyrine by the mouth in milder ones.[20] In the delirium of lobular pneumonia Bozzolo found injections of carbolic acid, or caffeine, of use.[21] The cold bath treatment has been praised by Rendu, and seventeen cases were successfully treated by iced compresses by W. F. Jackson, of Ontario, who confirmed Niemeyer's favourable account of this method.[22] He had previously reported twenty-five others, and in a useful discussion of the subject at Philadelphia showed a death-rate in 74 cases of only 2·7 per cent.[23] Mays believes that it checks the vascular stasis in the lungs, prevents exudation, and causes a rapid fall of temperature, pulse, and respiration. The value of calcium chloride in pneumonia has been explained by Crombie as being due to its neutralising the poisonous albumoses produced by the microbes, which otherwise would remove from the tissues the calcium salts necessary for life through the strong affinity which albumoses have for these calcium derivatives. Two fresh cases in which it was used with apparent benefit have been given in detail by Moir.[24]

**Asthma.**—A valuable paper by Markham Skerritt points out the use of caffeine as an agent of great power in relaxing pulmonary spasm.[25] It is one of the most reliable remedies we possess in the asthma of adults, especially in the form where attacks occur in the early morning. Five grains may be given every four hours when a paroxysm is present, otherwise five or ten grains may be administered at bedtime, and repeated if an attack comes on. In acute respiratory diseases where there is cardiac failure its effect also in stimulating the heart is of great value. Thorowgood[26] employs iodide of ethyl in spasmodic asthma as well as in fibroid phthisis. Six or eight drops may be inhaled from a piece of lint. Attacks may be aborted also by spraying the back with methyl chloride. This may be continued over the upper part of the chest if necessary.[27] O. Metcalfe has related some surprising cases of relief by massage of the chest muscles He finds a more or less tender area of the chest wall, on which he exerts pressure with rotatory movements of the finger-tips. After some twenty minutes of the manipulation great relief and very free expectoration follows.[28]

**Pleurisy.**—A most remarkable case where a serous effusion had remained eighteen months in the pleura, and was then tapped thirty-seven times without becoming purulent, is given by S. West.[29] The lung expanded completely, and the general health improved so that ultimate recovery might have been expected. However, a free incision was at last made, but the discharge became purulent, and a state of hectic fever ensued. This gradually lessened, and the patient regained good health, in spite of a phthisical family history. In another case, with an increasing effusion a negative pressure of four inches was observed, and air gained access to the pleura. Three days after, a positive pressure was noted, and ninety-seven ounces of clear fluid were withdrawn. There was no recurrence, and the air was rapidly re-absorbed.[30] Sutherland held that this great negative pressure was due to the fact that the elasticity of the lung was not exhausted, as is the case in the first stage of effusion; but West pointed out that this negative pressure diminishes very rapidly with the increase of fluid, and the amount in this case was extraordinary.

Ægophony was regarded by F. Taylor as merely a discord or dissonance produced by changes in the tubes resonating the higher harmonics alone, and not by a layer of fluid cutting off the lower ones.[31] Maguire denied that it is a true discord, but rather a confusion of sounds of the same pitch and different timbre. The difficulty of diagnosis of effusion in which ægophony and other signs so often fail is best met according to Whitney,[32] by noting the presence of the special curves of small, moderate, and large effusions, since these do not coincide with the outlines of consolidation; especially is this true of the S-shaped curve of moderate effusion. Cardiac dulness, too, extending to or beyond the midsternal line, is of early importance in left-sided effusions. Too little attention is often paid to these points. The absorption of effusion after exploratory punctures may be due to the stimulus of the irritation, at least Jordan and others have noted an increase of urine following such punctures.[33]

The treatment of dry pleurisy by injection of thirty drops of sterilised oil with a view to provide a lubricating fluid for the inflamed pleuræ has been tested by Cérenville, who found it[34] produced in some cases very satisfactory results. For ordinary empyœma Cumston has advocated the use of Revilliod's syphon. This tube has a bulb like a rectal injection pipe and produces continuous aspiration from the pleura, and has been successfully employed instead of a free incision and resection of the rib.[35] In this country the latter treatment has been generally accepted, but, as pointed out by J. H. Morgan,[36] its advantages over other methods are doubtful in the case of infants, empyæmas in chronic phthisis, and those which have opened into a bronchus. Collections of pus in both pleuræ are now, too, treated by incision with due precautions.

Subphrenic abscess has been the subject of papers by Laache, Adami, Buttersack, Hector Mackenzie, and F. C. Abbott. The two latter authors, believing that the epigastric swelling in their patient communicated freely with the pleura, drained it successfully by resecting the sixth rib.[37] In the discussion on this paper other cases were described which had been drained through the pleura, in some of them even when the pleura was healthy. In this way the risk of an abdominal opening was avoided. Laache found that about fifty cases of these abscesses have been published, and noticed the great difficulty of distinguishing the cases which communicate with the pleural cavity from the others.[38] He has operated both below and through the pleura, according to the position of the fluid. Buttersack[39] employed a double opening, but in his case the fluid burst through into the lung. Adami described a rapidly fatal case with distension of the pericardium, a cavity containing pus between it and the diaphragm, and another below the diaphragm communicating with the former.[40] The origin was found in a malignant ulceration of the stomach. Among other causes simple ulcers of the stomach, colon, and duodenum, hepatic abscess, and perityphlitis have been discovered.

[1] J. Path. and Bacteriol., April, 1895. [2] B.M.J., Feb. 9. [3] Amer. Journ. Med. Sc., Feb. [4] B.M.J., April 13. [5] B.M.J., Feb. 15. [6] B.M.J., Dec. 8, 1894. [7] B.M.J., Mar. 8, 1895. [8] Med. Week. Jan. 4. [9] Med. and S. Bullet., Jan. [10] Edin. Med. Journ., Dec., 1894. [11] Lancet, Jan. 19, 1895. [12] B.M.J., Mar. 2. [13] Fortsch. d. Med., Dec., 1894. [14] N.Y. Med. Journ., Feb. 23, 1895. [15] Lancet, April 20. [16] Amer. Journ. Med. Sc., Oct. 24, 1894. [17] Birmingh. Med. Rev., April, 1895. [18] Therap. Gaz., Jan. [19] Therap. Gaz., April. [20] B.M.J., Mar. 8. [21] Med. Week, Feb. 1. [22] Therap. Gaz., Nov., 1894. [23] N.Y. Med. Journ., Nov. 17. [24] Med. Chron., Dec. [25] Practit., April, 1895 [26] Med. Chron., Mar. [27] Clin. Journ., May. [28] B.M.J., Jan. 12. [29] B.M.J., April 27. [30] Lancet, Mar. 16 to April 6. [31] B.M.J., Feb. 16. [32] Med. Rec., Jan. 5. [33] Amer. Journ. Med. Sc., Jan. [34] Lancet, Dec. 1, 1894. [35] Therap. Gaz., Jan., 1895. [36] Clin. Journ., April 24. [37] Lancet, Nov. 3, 1894. [38] B.M.J., Jan. 13, 1895. [39] B.M.J., Mar. 16. [40] Practit., Oct., 1894.

